# A curious case of cutaneous elastolysis

**DOI:** 10.1016/j.jdcr.2025.09.037

**Published:** 2025-10-10

**Authors:** Robert J. Vanaria, Claudia Green, Allen Sapadin

**Affiliations:** a Hackensack Meridian School of Medicine, Nutley, New Jersey; b Department of Dermatology, Hackensack University Medical Center-Palisades, North Bergen, New Jersey

**Keywords:** dermal elastolysis, elastic tissue disorders, pseudoxanthoma elasticum

## Case description

**Fig 1:**
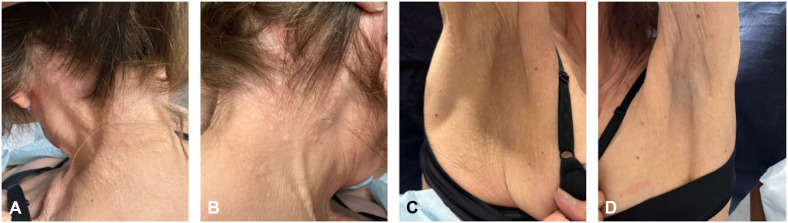
Asymptomatic-to-mildly pruritic skin lesions on the posterior neck and axillae.

A 76-year-old woman with no significant medical history presented with asymptomatic-to-mildly pruritic skin lesions on the back of neck and axillae (Fig 1), present for approximately 6 months. Physical examination revealed agminated yellow-white papules and nodules forming cobblestone-like plaques. The patient denied visual changes, hemoptysis, hematochezia, or cardiac symptoms. Her only notable history was hair dye use over 4 decades. A 4-mm punch biopsy from the right side of the back of the neck was collected for hematoxylin and eosin staining.

## Clinical questions

**Question 1: What is the most likely diagnosis?**

**A.** Pseudoxanthoma elasticum (PXE)
**B.** PXE-like papillary dermal elastolysis (PXE-PDE)
**C.** Mid-dermal elastolysis
**D.** White fibrous papulosis of the neck
**E.** Papular elastorrhexis

**Answers:**

A. Pseudoxanthoma elasticum (PXE) – Incorrect. PXE is an inherited disorder that presents in childhood and adolescence with similar skin findings but has systemic features (retinal angioid streaks, gastrointestinal bleeding, and coronary artery disease). Histology shows calcified elastic fibers.
B. PXE-like papillary dermal elastolysis (PXE-PDE) – Correct. Characterized by yellow-white papules on the neck and axillae, often asymptomatic, occasionally pruritic. Biopsy: papillary dermal elastic fiber loss on Shikata, no calcification. No systemic involvement, unlike PXE.^1^
C. Mid-dermal elastolysis – Incorrect. Presents with fine wrinkling or perifollicular papules on trunk or arms from loss of elastic fibers in the mid dermis.
D. White fibrous papulosis of the neck – Incorrect. Resembles PXE-PDE but shows collagen thickening on histology. Lesions are firm, white papules on the lateral aspect of the neck, lacking yellow hue or cobblestone pattern.
E. Papular elastorrhexis – Incorrect. Occurs in children or young adults with hypopigmented papules on trunk or extremities. Histology shows elastolysis but without cobblestone appearance or classic PXE-PDE distribution.

**Question 2: Which of the following histologic features best distinguishes PXE-PDE from PXE?**

**A.** Calcification of elastic fibers
**B.** Collagen thickening in the papillary dermis
**C.** Loss of elastic fibers in the mid dermis
**D.** Presence of dermal mucin
**E.** Fragmentation of elastic fibers without calcification

**Answers:**

A. Calcification of elastic fibers – Correct. Hallmark of inherited PXE, seen with special stains (eg, Von Kossa). PXE-PDE shows elastolysis without calcification.^2^
B. Collagen thickening in the papillary dermis – Incorrect. Characteristic of white fibrous papulosis of the neck with thickened papillary dermal collagen, no calcified fibers.^1^^,^^3^
C. Loss of elastic fibers in the mid dermis – Incorrect. Seen in mid-dermal elastolysis.
D. Presence of dermal mucin – Incorrect. Seen in connective tissue diseases (lupus and dermatomyositis).
E. Fragmentation of elastic fibers without calcification – Incorrect. Seen in papular elastorrhexis or PXE-PDE. Not specific for PXE.

**Question 3: Which of the following is true regarding PXE-PDE?**

**A.** It is associated with *ABCC6* mutations
**B.** It requires cardiovascular workup to assess for systemic involvement
**C.** It often presents in childhood or adolescence
**D.** It has no systemic associations
**E.** It commonly presents with mucosal ulcerations

**Answers:**

A. It is associated with *ABCC6* mutations – Incorrect. *ABCC6* mutations are seen in inherited PXE. PXE-PDE is not genetically linked.
B. It requires cardiovascular workup to assess for systemic involvement – Incorrect. PXE-PDE does not involve systemic organs.
C. It often presents in childhood or adolescence – Incorrect. PXE-PDE typically affects older women.
D. It has no systemic associations – Correct. PXE-PDE is an acquired cutaneous condition with no systemic involvement.
E. It commonly presents with mucosal ulcerations – Incorrect. Mucosal involvement is more suggestive of autoimmune blistering or ulcerative dermatoses.

Biopsy revealed lack of elastic fibers in the papillary dermis on Shikata staining, findings consistent with PXE-PDE.

## Discussion

PXE-PDE is a rare, acquired elastolytic disorder that predominantly affects elderly women. Clinically, it presents with asymptomatic or mildly pruritic yellow-white papules, often clustered into cobblestone-like plaques on the neck and flexural regions. Because the lesions closely resemble those of inherited PXE, distinguishing the 2 entities is essential. Unlike PXE, PXE-PDE is not associated with systemic manifestations such as ocular angioid streaks, gastrointestinal hemorrhage, or cardiovascular disease.

Histopathology is diagnostic. PXE-PDE demonstrates selective loss of elastic fibers in the papillary dermis without calcification, best highlighted with special elastic stains such as Verhoeff-Van Gieson or Shikata. In contrast, PXE shows calcified elastic fibers and systemic involvement due to *ABCC6* gene mutations. Other elastolytic disorders in the differential include white fibrous papulosis of the neck, mid-dermal elastolysis, papular elastorrhexis, and interstitial granuloma annulare, each with distinct clinical and histologic features.

Recognition of PXE-PDE is important to avoid unnecessary systemic evaluation and patient anxiety with misdiagnosis of PXE. Although its pathogenesis remains unclear, chronic actinic damage and intrinsic aging have been proposed as contributing factors. Management is largely conservative, with emphasis on reassurance and cosmetic counseling.

## Conflicts of interest

None disclosed.
